# Molecular Characterization of *Staphylococcus aureus* and Coagulase‐Negative Staphylococci Isolates at a Rural Hospital in Southern Malawi

**DOI:** 10.1155/bmri/2646451

**Published:** 2026-04-17

**Authors:** Wilfred Taika, Mulemba Tillika Samutela, Melissa Pender, Bernard Mudenda Hang′ombe

**Affiliations:** ^1^ Department of Paraclinical Studies, School of Veterinary Medicine, University of Zambia, Lusaka, Zambia, unza.zm; ^2^ Department of Biomedical Sciences, Malamulo College of Health Sciences, Malawi Adventist University, Makwasa, Malawi; ^3^ Department of Biomedical Sciences, School of Health Sciences, University of Zambia, Lusaka, Zambia, unza.zm; ^4^ Department of Medicine, Malamulo Adventist Hospital, Makwasa, Malawi; ^5^ Department of Medicine, School of Medicine, Loma Linda University Health, Loma Linda, California, USA, lluh.org

**Keywords:** antimicrobial resistance, coagulase-negative staphylococci, Malawi, resistance genes, *spa* typing, *Staphylococcus aureus*, virulence genes

## Abstract

**Objectives:**

This study is aimed at identifying antimicrobial resistance (AMR) and virulence‐encoded genes in *Staphylococcus aureus* and coagulase‐negative staphylococci (CoNS) isolates from a rural hospital in Southern Malawi. *S*. *aureus* isolates were further characterized by typing of staphylococcal protein A (*spa*).

**Methods:**

We conducted a cross‐sectional study of 36 *Staphylococcus* isolates from clinical samples collected at Malamulo Hospital in Makwasa, Malawi. Antimicrobial susceptibility testing was performed using the disc diffusion method. Polymerase chain reaction (PCR) was used to detect AMR genes (*mec*A*, erm*A*, erm*B*, erm*C*, tet*K*, tet*L*, tet*M, and *tet*O) and virulence genes (PVL: *lukS-PV, lukF-PV*; *spl*A*, spl*B*, spl*C*, spl*D*, spl*E, and *spl*F). PCR also confirmed *S. aureus* isolates through the detection of *nuc*, *spa,* or *coa* genes. *Spa* typing of *S. aureus* isolates was performed by Sanger sequencing.

**Results:**

The 36 isolates were identified as 14 *S*. *aureus* and 22 CoNS species. The highest resistance among all isolates was against sulfamethoxazole/trimethoprim (*n* = 18, *χ* ^2^ = 2.338, df = 2, *p* = 0.311), penicillin (*n* = 15, *χ* ^2^ = 2.258, df = 1, *p* = 0.175), and tetracycline (*n* = 14, *χ* ^2^ = 1.190, df = 1, *p* = 0.314). One CoNS and three *S. aureus* isolates demonstrated phenotypic multidrug methicillin resistance. The *tet*K gene was significantly more prevalent among *S. aureus* compared with CoNS isolates (*χ* ^2^ = 10.227, df = 1, *p* < 0.01). No significant difference was found between *S. aureus* and CoNS in the detection of *mec*A (*χ* ^2^ = 1.063, df = 1, *p* = 0.547), *tet*L (*χ* ^2^ = 1.348, df = 1, *p* = 0.511), *tet*M (*χ* ^2^ = 0.110, df = 1, *p* = 1.000), or PVL genes (*lukS-PV* and *lukF-PV*) (*χ* ^2^ = 4.129, df = 1, *p* = 0.064)*. Spa* typing identified seven *spa* types, with the most common type identified as t941 (*n* = 3).

**Conclusion:**

This study reveals the presence of both multidrug‐resistant CoNS and *S. aureus* strains with diverse *spa* types in rural Southern Malawi and highlights a role for molecular testing in surveillance and diagnostic testing.

## 1. Introduction

*Staphylococcus aureus* and coagulase‐negative staphylococci (CoNS) are common pathogens worldwide, known for their potential to develop antimicrobial resistance (AMR) and risk for severe systemic disease [1–4]. In 2021, *S. aureus* caused an estimated 325,165 global deaths, with methicillin‐resistant *Staphylococcus aureus* (MRSA) contributing significantly to mortality due to multidrug resistance [4]. Africa faces a disproportionate burden, with MRSA rates up to 40.8%, and East Africa reporting a 19% prevalence [5]. West and East Africa may be hotspots for the dissemination of antimicrobial resistance genes (ARGs) in *S. aureus*, fueled by excessive antibiotic use and inadequate infection control at tightly linked human, animal, and environmental ecosystems [6–9]. In Malawi, MRSA has been increasingly reported, rising from 7.7% in 1998 to 18.4% of *S. aureus* isolates by 2016 [10]. *S*. *aureus* is among the leading bacterial causes of pediatric mortality and one of the most frequently identified pathogens in Malawian tertiary hospitals [11–13]. The CoNS species are increasingly recognized as nosocomial pathogens, particularly among immunocompromised patients, with multidrug‐resistant strains commonly isolated [14]. Between 2017 and 2022, the Malawi Ministry of Health developed a National Action Plan on AMR, which established sentinel sites for AMR monitoring across animal and human populations [15–17]. Despite surveillance efforts, AMR data remain scarce due to inadequate funding, lack of trained personnel, poor infrastructure, and limited data storage among health facilities [18]. The AMR surveillance sites in Malawi often lack the capacity to perform molecular analyses of local bacterial strains [13, 19]. Additionally, much of the AMR data from Malawi results from urban tertiary rather than rural facilities [11, 13, 20–24]. As a result, treatment and prevention efforts are hindered by limited data on the molecular characteristics of regional staphylococci strains [4, 19].

Molecular surveillance of regional staphylococcal strains remains essential for rapid detection and early intervention [4, 25]. Polymerase chain reaction (PCR) enables the detection and amplification of genes encoding resistance and virulence. Panton–Valentine leukocidin (PVL) and serine protease–like (Spl) proteins are virulence factors that modulate the pathogenicity and immunogenicity of *S. aureus* strains [26, 27]. Sequencing the polymorphic X region of the *S. aureus* protein A gene (spa typing) serves as a biosensor that aids epidemiological surveillance by facilitating outbreak investigations [28, 29]. Genes encoding AMR and virulence are potential targets for diagnostic and therapeutic interventions [4, 30]. This study investigated the molecular patterns of AMR and virulence markers among *S. aureus* and CoNS isolates from clinical samples collected at Malamulo Hospital, a rural referral center and Fleming Fund AMR surveillance site [18] in Southern Malawi. We are aimed at improving the understanding of regional AMR profiles which may assist in the development of local treatment protocols.

## 2. Materials and Methods

### 2.1. Study Design and Sites

This cross‐sectional study analyzed 36 *Staphylococcus* isolates from clinical specimens collected at Malamulo Hospital between December 2022 and May 2023. All nonredundant staphylococci species confirmed by the laboratory information system (LIS) were included. Redundant positive samples and nonstaphylococci isolates, as confirmed by the LIS, were excluded from analysis. Clinical and biochemical identification and phenotypic resistance testing were completed at Malamulo Hospital before specimen transfer to the University of Zambia in Lusaka, Zambia, for molecular characterization.

### 2.2. Specimen Collection

Staphylococcal isolates were collected from patients with systemic or local infections during the study period. We documented patient demographics, specimen type, and inpatient versus outpatient clinical setting. Specimens included venous blood (*n* = 16), urine (*n* = 14), purulent fluid (*n* = 3), ascitic fluid (*n* = 1), pleural fluid (*n* = 1), and skin tissue biopsy (*n* = 1).

### 2.3. Biochemical Identification of Staphylococcal Isolates

Specimens were inoculated on a nutrient agar plate, incubated for 24 h at 37°C, and identified using Gram staining, catalase, and slide coagulase tests. *S*. *aureus* and CoNS were differentiated by a positive vs. negative coagulase test, respectively.

### 2.4. Antimicrobial Susceptibility Testing using Disc Diffusion Methods

Phenotypic antimicrobial susceptibility was tested for all isolates using the Kirby–Bauer disc diffusion method (BD BBL Sensi‐Disc Antimicrobial Susceptibility Test, Becton Dickinson) on Mueller–Hinton agar. The following antimicrobial susceptibility test discs were applied according to manufacturer′s protocols: erythromycin (15 *μ*g), penicillin (1 unit), ciprofloxacin (5 *μ*g), sulfamethoxazole/trimethoprim (23.75/1.25 *μ*g), gentamicin (10 *μ*g), cefoxitin (30 *μ*g), and tetracycline (30 *μ*g). After 24 h of incubation at 35°C, the zone of inhibition was measured in millimeters. The reference strains *S. aureus* ATCC 29213 and *Staphylococcus epidermidis* ATCC 12228 were used as quality control organisms for antimicrobial susceptibility testing of the *S. aureus* and CoNS isolates, respectively. Resistance was interpreted using European Committee on Antimicrobial Susceptibility Testing (EUCAST) breakpoints [28]. Multidrug resistance was defined as resistance to at least three antibiotic classes [29].

### 2.5. DNA Extraction and Purification

Staphylococcal DNA was extracted from all isolates using the boiling method [30], with pure DNA defined as an absorbance ratio (A260/A280) of at least 1.8 ng/*μ*L DNA concentration. Isolates were stored in 25% glycerol at −80°C for the duration of the study period.

### 2.6. PCR Identification of ARGs

PCR amplified genes encoding resistance to methicillin (*mec*A), erythromycin (*erm*A, *erm*B, and *erm*C), and tetracycline (*tet*K, *tet*L, *tet*M, and *tet*O) from all isolates using standardized PCR primers and amplification conditions (Table 1) [32–35]. Expected PCR product base pair lengths were 533 bp (*mecA*), 645 bp (*ermA*), 639 bp (*ermB*), 642 bp (*ermC*), 616 bp (*tetK*), 456 bp (*tetL*), 576 bp (*tet*M), and 515 bp (*tet*O).

**Table 1:** Primers and PCR conditions.

Gene	Forward primer	Reverse primer	Components	Steps	Temp. (°C)	Time (Min.)	Cycles	Reference
*nuc*	5 ^′^ ‐ GCGATTGATGGT GAT ACGGTT – 3 ^′^	5 ^′^ ‐ AGCCAAGCCTTG ACGAACTAA AGC – 3 ^′^	10‐*μ*L master mix (4.8 − *μ*L master mix with standard buffer + 0.2 *μ*L of each 10 − *μ*M primer + 2.8 − *μ*L nuclease free water + 2 − *μ*L template DNA)	Initial denaturation	94	5	1×	[31]
				Denaturation	94	1	30×	
				Annealing	50	1		
				Extension	72	2		
				Final extension	72	10	1×	
*coa*	5 ^′^ – GTAGATTGGGCA ATTACA TTTGG AGG– 3 ^′^	5 ^′^ – CGCATCAGCTTT GTTATCCCATGTA – 3 ^′^	10‐*μ*L master mix (4.8 − *μ*L master mix with standard buffer + 0.2 − *μ*L of each 10 − *μ*M primer + 2.8 − *μ* nuclease free water + 2 − *μ*L template DNA)	Initial denaturation	94	4	1×	[31]
				Denaturation	94	0.45	30×	
				Annealing	50	0.45		
				Extension	72	1		
				Final extension	72	2	1×	
*spa*	5 ^′^ ‐ AGACGATCCTTCGGTGAGC – 3 ^′^	5 ^′^‐ GCTTTTGCAATGTCATTTACTG – 3 ^′^	25‐*μ*L master mix (12.5 − *μ*L master mix with standard buffer + 0.5 − *μ*L of each 10 − *μ*M primer + 7 − *μ*L nuclease free water + 5 − *μ*L template DNA)	Initial denaturation	95	4	1×	[31]
				Denaturation	95	0.30	30×	
				Annealing	60	0.30		
				Extension	72	0.45		
				Final extension	72	10	1×	
*mec*A	5 ^′^‐AAAATCGATGGTAAAGGTTGGC‐3 ^′^	5 ^′^‐AGTTCTGCAGTACCGGATTTGC‐3 ^′^	10‐*μ*L master mix (4.8 − *μ*L master mix with standard buffer + 0.2 − *μ*L of each 10 − *μ*M primer + 2.8 − *μ* nuclease free water + 2 − *μ*L template DNA)	Initial denaturation	94	4	1×	[32]
				Denaturation	94	0.30	30×	
				Annealing	53	0.30		
				Extension	72	1		
				Final extension	72	4	1×	
*erm*A	5 ^′^‐ TCTAAAAAGCATGTAAAAGAA‐3 ^′^	5 ^′^‐ CTTCGATAGTTTATTAATATTAG‐3 ^′^	10‐*μ*L master mix (4.8 − *μ*L master mix with standard buffer + 0.2 − *μ*L of each 10 − *μ*M primer + 2.8 − *μ* nuclease free water + 2 − *μ*L template DNA)	Initial denaturation	93	3	1×	[33, 34]
*erm*B	5 ^′^‐ GAAAAGTACTCAACCAAATA‐3 ^′^	5 ^′^‐ AGTAACGGTACTTAAATTGTTTA‐3 ^′^		Denaturation	93	1	30×	
*erm*C	5 ^′^‐ TCAAAACATAATATAGATAAA ‐3 ^′^	5 ^′^‐ GCTAATATTGTTTAAATCGTCAAT ‐3 ^′^		Annealing	52	1		
				Extension	72	1		
				Final extension	72°C	5	1×	
*tet*K	5 ^′^‐ TTAGGTGAAGGGTTAGGTCC ‐3 ^′^	5 ^′^‐ GCAAACTCATTCCAGAAGCA ‐3 ^′^	10‐*μ*L master mix (4.8 − *μ*L master mix with standard buffer + 0.2 − *μ*L of each 10 − *μ*M primer + 2.8 − *μ* nuclease free water + 2 − *μ*L template DNA)	Initial denaturation	93	3	1×	[35]
*tet*L	5 ^′^‐ CATTTGGTCTTATTGGATCG ‐3 ^′^	5 ^′^‐ ATTACACTTCCGATTTCGG ‐3 ^′^		Denaturation	93	1	30×	
				Annealing	52	1		
*tet*M	5 ^′^‐ GTTAAATAGTGTTCTTGGAG‐3 ^′^	5 ^′^‐ CTAAGATATGGCTCTAACAA‐3 ^′^		Extension	72	1		
*tet*O	5 ^′^ – GATGGCATACAGGCACAGAC‐3 ^′^	5 ^′^ – CAATATCACCAGAGCAGGCT‐3 ^′^		Final extension	72	5	1×	
PVL	5 ^′^ ‐ GCTGGACAAAACTTCTTGGAATAT – 3 ^′^	5 ^′^ ‐ GATAGGACACCAATAAATTCTGGATTG – 3 ^′^	10‐*μ*L master mix (4.8 − *μ*L master mix with standard buffer + 0.2 − *μ*L of each 10 − *μ*M primer + 2.8 − *μ* nuclease free water + 2 − *μ*L template DNA)	Initial denaturation	94	5	1×	[26]
				Denaturation	94	0.30	30×	
				Annealing	59	1		
				Extension	72	1		
				Final extension	72	10	1×	
*spl*A	5 ^′^‐CATTCAATTGCCGGATCCGAAAAGAATGTC‐3 ^′^	5 ^′^‐CACGAATGAATTGACTCGAGTTATTTTTCAATAT‐3 ^′^	10‐*μ*L master mix (4.8 − *μ*L master mix with standard buffer + 0.2 − *μ*L of each 10 − *μ*M primer + 2.8 − *μ* nuclease free water + 2 − *μ*L template DNA)	Initial denaturation	95	2	1×	[27]
*spl*B	5 ^′^‐CAACAAACTGCCGGATCCGAAAATAATGTC‐3 ^′^	5 ^′^‐GCTCGTTTAAAGTCACTCGAGTTATTTATCTATG‐3 ^′^		Denaturation	95	0.30	30×	
*spl*C	5 ^′^‐GGATCCGAGAAGAATGTTACGCAAGTTAAAG‐3 ^′^	5 ^′^‐ CTCGAGTTATTGTTCAATGTGCTTTTGAATAAAATC‐3 ^′^		Annealing	48	0.30		
*spl*D	5 ^′^‐ GGATCCGAAAATAGTGTGAAATTAATTACCAACACG‐3 ^′^	5 ^′^‐ CTCGAGTTATTTATCTAAATTATCTGCAATAAATTTC‐3 ^′^		Extension	72	5		
*spl*E	5 ^′^‐ GGATCCGAACATAATGTGAAACTAATCAAAAATAC‐3 ^′^	5 ^′^‐ CTCGAGTTATTTATCTGTGTTATCTGCAATGAATTTC‐3 ^′^		Final extension	72	5	1×	
*splF*	5 ^′^‐ CAACAAACAGCCGGATCCGAAAATACTGTTAAAC‐3 ^′^	5 ^′^‐ GTCTAAGCTCGTGTTATTTATCTAAATTATC‐3 ^′^	Same as in *spl*E above	Same as in *spl*E above	Same as in *spl*E above	Same as in *spl*E above	Same as in *spl*E above	[27]

### 2.7. PCR Identification of Virulence Genes

Staphylococcal virulence genes among all isolates were identified using gene‐specific primers and PCR conditions for PVL (*lukS-PV* and *lukF-PV*), *spl*A, *spl*B, *spl*C, *spl*D, *spl*E, and *spl*F genes (Table 1). Expected PCR product base pair lengths were 83 bp (*lukS-PV* and *lukF-PV*), 641 bp (*spl*A), 654 bp (*spl*B), 621 bp (*spl*C), 635 bp (*spl*D), 633 bp (*spl*E), and 621 bp (*spl*F) [26, 27].

### 2.8. PCR Identification and Confirmation of *S. aureus* Isolates

*S*. *aureus* isolates underwent PCR targeting the *nuc*, *coa*, and *spa* genes (Table 1) [35]. Isolates with detection of at least one of these targets were confirmed as *S. aureus*. The positive control for *nuc* and *coa* PCR assays was *S*. *aureus* ATCC 29213. Expected PCR product base pair lengths were 279 bp (*nuc* gene), 117 bp (*coa* gene), and 250–500 bp (*spa* gene).

### 2.9. *Spa* Typing of *S. aureus* Isolates Using Sanger Sequencing

The *Spa* gene PCR products from *S. aureus* isolates underwent BigDye Terminator sequencing [35] using the Seq Studio Genetic Analyzer (Applied Biosystems, Thermo Fisher Scientific Inc., Massachusetts, United States). Consensus sequences were assembled and aligned using Genetyx ATCG software (www.genetyx.co.jp) and exported in FASTA format. These sequences were analyzed using the NCBI Basic Local Alignment Search Tool (BLAST) (www.ncbi.nlm.nih.gov) to identify highly similar sequences. The *Spa* types were matched using the following online tools: spaTypeFinder (spatyper.fortinbras.us) and spaTyper from the Center for Genomic Epidemiology (genomicepidemiology.org).

### 2.10. Data Analysis

Chi‐square and Fisher′s exact tests were used to analyze the data, with statistical significance set at *p* < 0.05. The chi‐square test was used for comparing proportions in categorical variables when expected cell counts were five or more. Fisher′s exact test was applied when expected cell counts were below five.

## 3. Results

### 3.1. Patient Demographics and Biochemical Identification of Staphylococcal Isolates

Isolates were obtained from 36 pediatric and adult patients, consisting of 14 males (39%) and 22 females (61%) (Table 2). The majority were inpatients (*n* = 29) compared with outpatients (*n* = 7). Slide coagulase testing differentiated 14 *S. aureus* isolates from 22 CoNS isolates. *S*. *aureus* was detected among inpatients more frequently than CoNS (*χ* ^2^ = 5.530, df = 1, *p* < 0.05), but there were no significant differences in identification based on specimen type (*χ* ^2^ = 6.708, df = 5, *p* = 0.167) or patient gender (*χ* ^2^ = 0.564, df = 1, *p* = 0.501).

**Table 2:** Patients′ demographic information.

	*N* = 36	(%)	CoNS (*n* = 22)	*S. aureus* (*n* = 14)	^*X*2^ (df)	p value	Phi
Age categories					4.7 (3)	0.150	0.361
Pediatric (0–14 years old)	12	33	8	4			
Youth group (15–47 years old)	19	53	13	6			
Middle aged (48–63 years old)	2	6	0	2			
Elderly (≥ 64 years old)	3	8	1	2			
Patients′ sex					0.564 (1)	0.501	−0.125
Male	13	36	9	4			
Female	23	64	13	10			
Type of care∗					5.530 (1)	0.029	−0.392
Outpatient care	7	19	7	0			
Inpatient care	29	81	15	14			
Took antibiotic(s) within the past 7 days					1.616 (1)	0.389	0.212
Yes (benzylpenicillin, gentamicin)	1	3	0	1			
No	35	97	22	13			
Type of specimen collected					6.708 (5)	0.167	0.432
Ascitic fluid	1	3	0	1			
Pleural fluid	1	3	1	0			
Skin biopsy	1	3	0	1			
Pus	3	8	1	2			
Urine	14	39	14	3			
Venous blood	16	44	9	7			

*Note:* *p* values chi‐square (*χ* ^2^) or Fisher′s exact test was used as appropriate.

^∗^Groups are significantly different (*p* < 0.05).

### 3.2. Antimicrobial Susceptibility

There were no significant differences between the phenotypic resistance profiles of *S. aureus* and CoNS isolates (Table 3). Resistance was highest against sulfamethoxazole/trimethoprim (*n* = 18, *χ* ^2^ = 2.338, df = 2, *p* = 0.311), penicillin (*n* = 15, *χ* ^2^ = 2.258, df = 1, *p* = 0.175), and tetracycline (*n* = 14, *χ* ^2^ = 1.190, df = 1, *p* = 0.314). Erythromycin demonstrated moderate resistance (*n* = 11, *χ* ^2^ = 3.358, df = 2, *p* = 0.187) whereas the lowest resistance rates were noted for gentamicin (*n* = 5, *χ* ^2^ = 1.089, df = 1, *p* = 0.357), cefoxitin (*n* = 6, *χ* ^2^ = 0.374, df = 1, *p* = 0.658), and ciprofloxacin (*n* = 7, *χ* ^2^ = 1.218, df = 1, *p* = 0.394). Four isolates (three *S. aureus* and one CoNS) demonstrated multidrug resistance.

**Table 3:** Phenotypic and genotypic antimicrobial susceptibility profiles, spa types, and virulence genes of the isolates.

**a. Phenotypic antimicrobial susceptibility profile**							
	**N** = 36	**(%)**	**CoNS (** **n** = 22 **)**	***S. aureus* (n = 14)**	^***X*2**^ **(df)**	**p value**	**Phi**
Sulfamethoxazole/trimethoprim					2.338 (2)	0.311	0.255
Resistant	18	50	9	9			
Intermediate	2	6	1	1			
Sensitive	16	44	12	4			
Penicillin					2.258 (1)	0.175	−0.250
Resistant	15	42	7	8			
Sensitive	21	58	15	6			
Tetracycline					1.190 (1)	0.314	−0.182
Resistant	14	39	7	7			
Sensitive	22	61	15	7			
Erythromycin					3.358 (2)	0.187	0.305
Resistant	11	31	5	6			
Intermediate	3	8	1	2			
Sensitive	22	61	16	6			
Gentamicin					1.089 (1)	0.357	−0.174
Resistant	5	14	2	3			
Sensitive	31	86	20	11			
Cefoxitin					0.374 (1)	0.658	−0.102
Resistant	6	17	3	3			
Sensitive	30	83	19	11			
Ciprofloxacin					1.n (1)	0.394	−0.184
Resistant	7	19	3	4			
Sensitive	29	81	19	10			

**b. Molecular identification, confirmation, and spa typing of *Staphylococcus aureus* species**							
*nuc* gene detection ^∗∗^					21.758 (1)	< 0.001	0.777
Positive	10	28	0	10			
Negative	26	72	22	4			
*coa* gene detection ^∗∗^					9.124 (1)	0.005	0.503
Positive	5	14	0	5			
Negative	31	86	22	9			
*spa* gene detection ^∗∗^					18.857 (1)	< 0.001	0.724
Positive	9	25	0	9			
Negative	27	75	22	5			
spa types ^∗^					18.857 (7)	0.009	0.724
t941	3	8.3	0	3			
t6140	1	2.8	0	1			
t1130	1	2.8	0	1			
t021	1	2.8	0	1			
t002	1	2.8	0	1			
t064	1	2.8	0	1			
Unknown (Repeat Succession 11‐19‐12‐05‐17‐34‐24‐34‐22)	1	2.8	0	1			
Negative	27	75	22	5			

**c. Detection of antimicrobial resistance genes**							
	**N** = 36	**(%)**	**CoNS (n = 22)**	***S. aureus* (n = 14)**	^***X*2**^ **(df)**	**p value**	**Phi**
*mec*A gene detection					1.063 (1)	0.547	0.172
Positive	3	8	1	2			
Negative	33	92	21	12			
*erm*A gene detection					0.655 (1)	1.000	−0.135
Positive	1	3	1	0			
Negative	35	97	21	14			
*tet*K gene detection ^∗∗^					10.227 (1)	0.003	0.533
Positive	8	22	1	7			
Negative	28	78	21	7			
*tet*L gene detection					1.348 (1)	0.511	−0.193
Positive	2	6	2	0			
Negative	34	94	20	14			
*tet*M gene detection					0.110 (1)	1.000	0.055
Positive	2	6	1	1			
Negative	34	94	21	13			
*tet*O gene detection					3.328 (1)	0.144	0.304
Positive	2	6	0	2			
Negative	34	94	22	12			

d. Detection of virulence genes							
PVL (*lukS-PV* and *lukF-PV*) detection					4.129 (1)	0.064	0.339
Positive	5	14	1	4			
Negative	31	86	21	10			
*spl*A gene detection ^∗∗^					13.655 (1)	< 0.001	0.616
Positive	7	19	0	7			
Negative	29	81	22	7			
*spl*B gene detection ^∗∗^					9.124 (1)	0.005	0.503
Positive	5	14	0	5			
Negative	31	86	22	9			
*spl*D gene detection ^∗∗^					10.227 (1)	0.003	0.533
Positive	8	22	1	7			
Negative	28	78	21	7			
*spl*E gene detection					3.328 (1)	0.144	0.304
Positive	2	6	0	2			
Negative	34	94	22	12			
*spl*F gene detection					3.328 (1)	0.144	0.304
Positive	2	6	0	2			
Negative	34	94	22	12			

*Note:* *p* values chi‐square (*χ* ^2^) or Fisher′s exact test was used as appropriate.

^∗^Groups are significantly different (*p* < 0.05).

^∗∗^Groups are significantly different (*p* < 0.005).

### 3.3. Detection of ARGs

**Figure 1:**
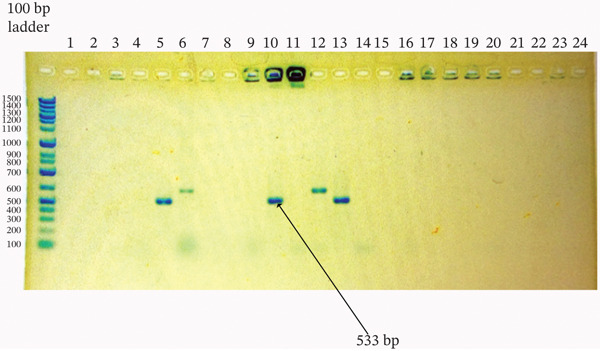
Agarose gel electrophoresis of *mec*A gene PCR products from *Staphylococcus aureus* and CoNS isolates. Amplified products were resolved on a 1.5% agarose gel stained with ethidium bromide. Ladder: 100‐bp DNA ladder; Lanes 5, 10, and 13: isolates positive for the target gene; Lanes 2–4, 6–9, 11, 12, and 14–24: negative isolates; Lane 1: no‐template control. The expected product size was ~533 bp.

**Figure 2:**
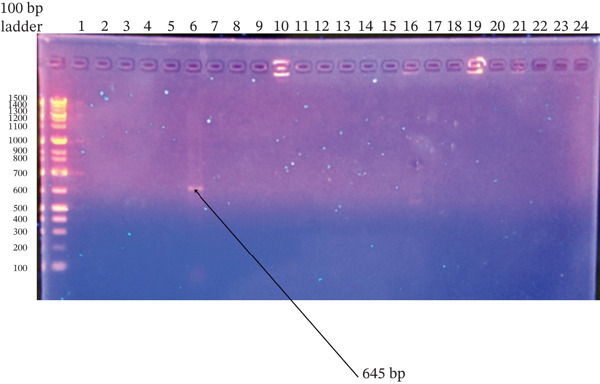
Agarose gel electrophoresis of *erm*A gene PCR products from *Staphylococcus aureus* and CoNS isolates. Amplified products were resolved on a 1.5% agarose gel stained with ethidium bromide. Ladder: 100‐bp DNA ladder; Lane 6: isolates positive for the target gene; Lanes 2–5, and 7–24: negative isolates; Lane 1: no‐template control. The expected product size was ~645 bp.

**Figure 3:**
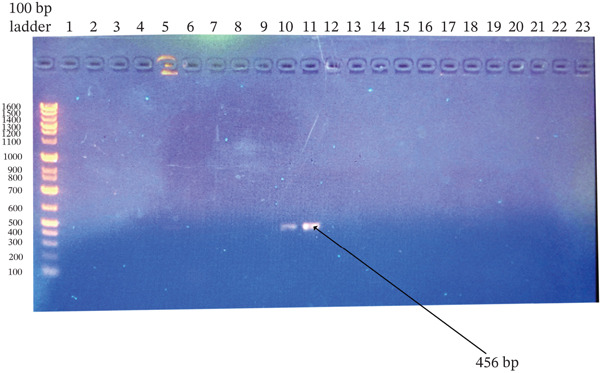
Agarose gel electrophoresis of the *tet*L gene PCR products from *Staphylococcus aureus* and CoNS isolates. Amplified products were resolved on a 1.5% agarose gel stained with ethidium bromide. Ladder: 100‐bp DNA ladder; Lanes 10 and 11: isolates positive for the target gene; Lanes 2–9 and 12–23: negative isolates; Lane 1: no‐template control. The expected product size was ~456 bp.

**Figure 4:**
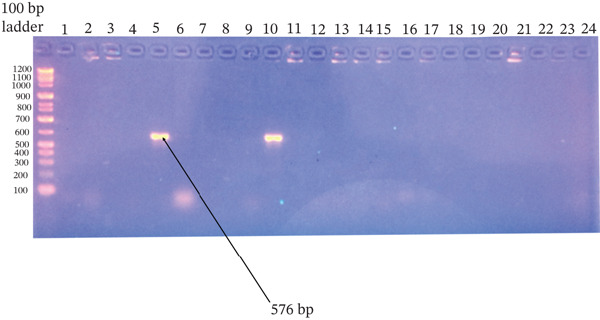
Agarose gel electrophoresis of the *tet*M gene PCR products from *Staphylococcus aureus* and CoNS isolates. Amplified products were resolved on a 1.5% agarose gel stained with ethidium bromide. Ladder: 100‐bp DNA ladder; Lanes 5 and 10: isolates positive for the target gene; Lanes 2–4, 6–9, and 11–24: negative isolates; Lane 1: no‐template control. The expected product size was ~576 bp.

The *tet*K gene was more commonly detected in *S. aureus* (*χ* ^2^ = 10.227, df = 1, *p* < 0.01) with a total of seven *S. aureus* and one CoNS isolate testing positive (Table 3). There was no significant difference between *S. aureus* and CoNS isolates in the detection of *mec*A (*χ* ^2^ = 1.063, df = 1, *p* = 0.547), *erm*A (*χ* ^2^ = 0.655, df = 1, *p* = 1.000), *tet*L (*χ* ^2^ = 1.348, df = 1, *p* = 0.511), and *tet*M genes (*χ* ^2^ = 0.110, df = 1, *p* = 1.000) (Figures 1, 2, 3, and 4).

### 3.4. Detection of Virulence Genes

**Figure 5:**
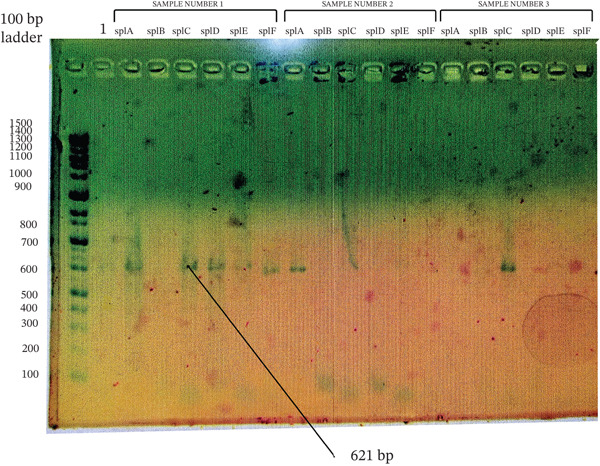
Agarose gel electrophoresis of *spl* genes PCR products from *Staphylococcus aureus*. Amplified products were resolved on a 1.5% agarose gel stained with ethidium bromide. Ladder: 100‐bp DNA ladder; Sample Number 1: *spl*A, *spl*C, *spl*D, *spl*E, and *spl*F positive for the target genes; Sample Number 2: *spl*A positive for the target gene; Sample Number 3: *spl*C positive for the target gene; Sample Number 1: *spl*B negative for the target gene; Sample Number 2: *spl*B, *spl*C, *spl*D, *spl*E, *spl*F negative for the target genes; Sample Number 3: *spl*A, *spl*B, *spl*D, *spl*E, and *spl*F negative for the target genes; Lane 1: no‐template control. The expected product sizes were 641 bp (*spl*A), 654 bp (*spl*B), 621 bp (*spl*C), 635 bp (*spl*D), 633 bp (*spl*E), and 621 bp (*spl*F).

**Figure 6:**
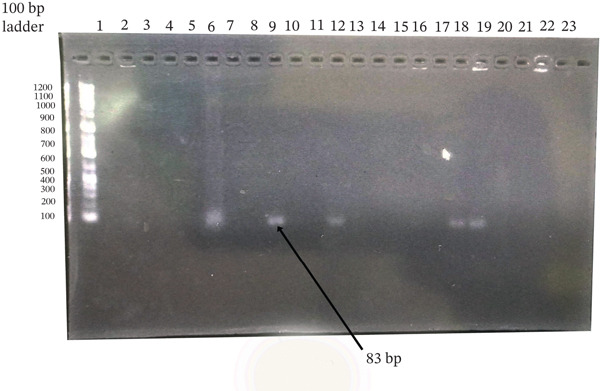
Agarose gel electrophoresis of PVL (*lukS-PV* and *lukF-PV* genes) PCR products from *Staphylococcus aureus* and CoNS isolates. Amplified products were resolved on a 1.5% agarose gel stained with ethidium bromide. Ladder: 100‐bp DNA ladder; Lanes 9, 12, 18, and 19: isolates positive for the target gene; Lanes 2–8, 10, 11, 13–17, and 20–23: negative isolates; Lane 1: no‐template control. The expected product size was ~83 bp.

The following virulence genes were more commonly detected among *S. aureus* compared with CoNS isolates: *spl*A (*χ* ^2^ = 13.655, df = 1, *p* < 0.01), *spl*B (*χ* ^2^ = 9.124, df = 1, *p* < 0.01), and *spl*D (*χ* ^2^ = 10.227, df = 1, *p* < 0.01) (Figure 5 and Table 3). There was no significant difference in the detection of the PVL genes *lukS-PV* and *lukF-PV* between *S. aureus* and CoNS isolates (*χ* ^2^ = 4.129, df = 1, *p* = 0.064) (Figure 6).

### 3.5. Molecular Identification, Confirmation, and *Spa* Typing of *S. aureus*

**Figure 7:**
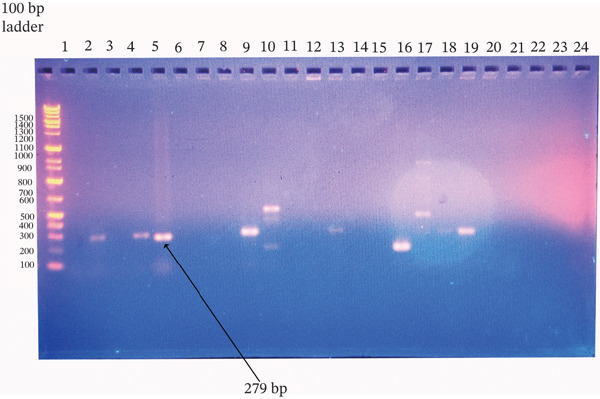
Agarose gel electrophoresis of *nuc* gene PCR products from *Staphylococcus aureus*. Amplified products were resolved on a 1.5% agarose gel stained with ethidium bromide. Ladder: 100‐bp DNA ladder; Lanes 2, 4, 5, 9, 13, 18, and 19: isolates positive for the target gene; Lanes 3, 6–8, 10–12, 14–17, and 20–24: negative isolates; Lane 1: no‐template control. The expected product sizes were ~279 bp.

**Figure 8:**
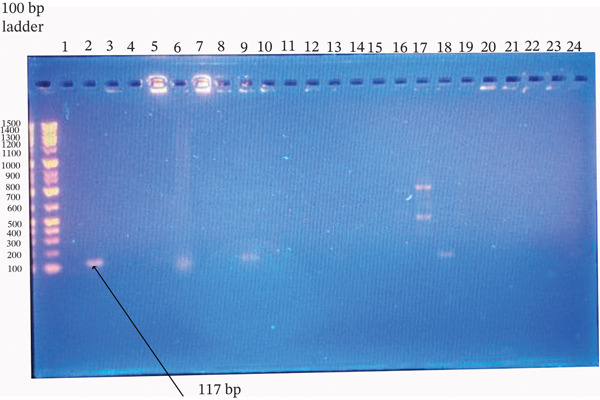
Agarose gel electrophoresis of the *coa* gene PCR products from *Staphylococcus aureus*. Amplified products were resolved on a 1.5% agarose gel stained with ethidium bromide. Ladder: 100‐bp DNA ladder; Lanes 2, 9, and 18: isolates positive for the target gene; Lanes 3–8, 10–17, and 19–24: negative isolates; Lane 1: no‐template control. The expected product size was ~117 bp.

**Figure 9:**
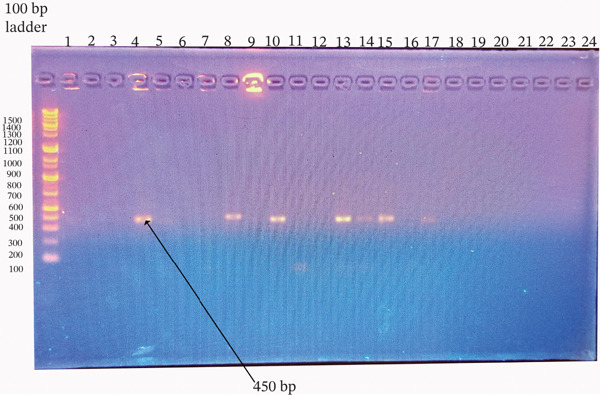
Agarose gel electrophoresis of *spa* gene PCR products from *Staphylococcus aureus*. Amplified products were resolved on a 1.5% agarose gel stained with ethidium bromide. Ladder: 100‐bp DNA ladder; Lanes 4, 8, 10, 13–15, and 17: isolates positive for the target gene; Lanes 2–3, 5–7, 9, 11, 12, 16, and 18–24: negative isolates; Lane 1: no‐template control. The expected product size was 250–500 bp.

The 14 *S. aureus* isolates were confirmed via PCR detection of the *nuc* gene (*n* = 10, *χ* ^2^ = 21.758, df = 1, *p* < 0.01; Figure 7), *coa* gene (*n* = 5, *χ* ^2^ = 9.124, df = 1, *p* < 0.01; Figure 8), and/or *spa* gene (*n* = 9, *χ* ^2^ = 18.857, df = 1, *p* < 0.01; Figure 9 and Table 3).

The most common *spa* type was t941 (*n* = 3) with other detected *spa* types including t6140, t1130, t021, t002, t064, and one unidentifiable *spa* type (Repeat Succession 11‐19‐12‐05‐17‐34‐24‐34‐22).

## 4. Discussion

In low‐income countries like Malawi, *Staphylococcus* identification often relies on conventional biochemical and culture‐based methods. Although these methods are often more accessible and cost‐effective than molecular testing, early detection of multidrug‐resistant organisms (MDROs) by routine methods is impeded by prolonged turnaround times and limited sensitivity when used in isolation [33]. Molecular techniques such as pulsed‐field gel electrophoresis, PCR‐restriction fragment length polymorphism, and multiplex PCR offer rapid and accurate identification of MDROs that can be adapted to resource‐limited settings [36, 37]. These tools support public health decision‐making by improving regional understanding of staphylococcal epidemiology [38]. Given regional variability in resistance patterns, treating staphylococcal infections remains challenging without molecular or culture‐based susceptibility data. Reported local resistance patterns help clinicians select empiric treatment [36]. Phenotypic *S. aureus* AMR findings in this study differ from regional findings from similar study settings. Marcelino et al., in Mozambique, reported resistance of *S. aureus* to penicillin (90%), tetracycline (48%), erythromycin (25%), and sulfamethoxazole/trimethoprim–resistance (11%) [37]. Shawa et al., in Zambia, reported resistance of *S. aureus* to penicillin (50%) [39], whereas Camara et al., in Tanzania, reported 82% *S. aureus* resistance rate to sulfamethoxazole/trimethoprim [40]. Sulfamethoxazole/trimethoprim–resistant *Staphylococcus* species have remained significant in Malawi. Bwanali *et al*. reported 83% sulfamethoxazole/trimethoprim–resistance, of which staphylococci species accounted for 62%, in Malawian public health facilities between 2021 and 2024 [41].

Although data on household usage and the HIV status of participants were not collected in this study, colonization with sulfamethoxazole/trimethoprim–resistant staphylococci strains among persons living with HIV has been reported in other African countries, including Kenya, 37% [42], and South Africa, 91% [43]. These reports suggest that daily sulfamethoxazole/trimethoprim prophylaxis use among patients on antiretroviral therapy may contribute to increasing sulfamethoxazole/trimethoprim–resistant staphylococci strains in regions with high prevalence of HIV. Daily sulfamethoxazole/trimethoprim has been shown to reduce HIV‐associated deaths in low‐income healthcare settings like Malawi [44]. Rising resistance rates decrease the effectiveness of sulfamethoxazole/trimethoprim for empiric treatment of bacterial infections in HIV‐endemic regions, warranting continued AMR surveillance and mitigation efforts [45]. Tetracycline use in Malawi has declined since the 1990s due to widespread resistance [46]; however, related antibiotics such as doxycycline continue to be used [47–49], and tetracycline itself is used for livestock on small‐scale farms [50, 51]. Given variations in both documented antibiotic use and AMR patterns in Southern Africa, continued empiric use of common antimicrobials without access to susceptibility data raises concern for unreliable and inappropriate antimicrobial treatment. Further regional studies with larger sample sizes are needed to inform local empiric treatment guidelines as well as to support the development of national and regional antibiograms to aid informed antibiotic selection [36]. As both *S. aureus* and CoNS share potential for AMR, all staphylococcal species warrant continued AMR surveillance. This study found no significant differences between *S. aureus* and CoNS in the detection of *mec*A, *erm*A, *tet*L, *tet*M, *tet*O, PVL genes, and *spl*D. These CoNS species are increasingly identified as nosocomial pathogens with notable AMR and cytotoxin production capable of causing serious infections, particularly among immunocompromised patients [15, 52].

In Malawi, a plethora of risk factors exist for hospital‐acquired multidrug‐resistant CoNS. Contributing factors include limitations in antimicrobial regulation, infection control, healthcare access, laboratory investigations, and organism identification, not to mention the impact of HIV/AIDS and malnutrition [16, 20, 53, 54]. In settings with poor diagnostic capacity, CoNS infections may be misidentified or overlooked as contaminants, leading to underestimation of their clinical impact [51]. Multidrug resistance among CoNS isolates commonly limits treatment choices and complicates infection control in hospital settings [52]. The most commonly isolated CoNS strains with methicillin resistance potential in Africa are *S*. *epidermidis* [53]. In this study, one methicillin‐resistant CoNS was detected, but further characterization and species identification of the CoNS isolate were not completed, as we had reserved these measures to target *S. aureus*. Future studies may wish to focus on the molecular characterization of CoNS in Malawi health facilities.

*Spa* typing allows classification of *S. aureus* strains to aid epidemiological analysis and outbreak tracking [28, 29]. The *spa* gene was detected in only 9 of the 14 *S. aureus* isolates. *S*. *aureus* was confirmed by detection of the *nuc*, *coa*, or *spa* gene, so the remaining 5 isolates were confirmed as *S. aureus* by detection of the *nuc* and/or *coa* gene. The most commonly identified *spa* type in this study was t941 (*n* = 3). The t941 strain was previously reported as a human origin methicillin‐susceptible *S. aureus* (MSSA) in Europe, Germany [54]. Among the t941 MSSA reported in this study, one was PVL (*lukS-PV* and *lukF-PV*) positive. The PVL‐positive *S. aureus* clonal complexes are considered to have sub‐Saharan African ancestry [55]. The t941 *spa* types reported in both Africa and Europe indicate that sustained local transmissions and population movements may be facilitating the spread and increase in diversity between the regions [56]. Due to scarce molecular data from rural Malawi, its origin remains uncertain, highlighting the need for expanded genomic surveillance to determine whether this strain represents local evolution or external introduction.

Reports from sub‐Saharan Africa have documented several of our identified spa types, specifically t002, t941, and t064, among patients with both asymptomatic carriage and invasive infection, including septic shock, endocarditis, and pneumonia [57, 58]. The remaining detected spa types are poorly reported and may represent regionally unique strains; further work is required to determine their clinical significance. Given that 11 isolates phenotypically demonstrated erythromycin resistance but *erm* expression was detected in only one isolate, we remain curious if resistance may be driven by other mechanisms, such as *mcr* or *mef* genes [59, 60]. Since *erm* genes typically mediate inducible resistance via ribosomal methylation, their limited occurrence indicates alternative pathways [57]. This highlights the importance of exploring additional resistance genes, including *mef (A)*, which can produce low‐level resistance in staphylococci even without detectable *erm* activity [58]. Furthermore, of the three *S. aureus* isolates with phenotypic methicillin resistance, *mec*A was detected from only two isolates. This observation suggests the potential involvement of alternative resistance mechanisms, such as *mecC*, other unknown genetic elements, or adaptations like altered penicillin‐binding protein (PBP) expression in the third isolate [61, 62]. This study has multiple limitations. Firstly, the small, heterogeneous sample size from a rural single‐center admittedly limits generalizability and introduces risk of bias due to sampling limitations. Secondly, our laboratory′s limited capacity prevented a comprehensive assessment of *S. aureus* resistance. We were unable to perform MIC testing, evaluate inducible tetracycline resistance, or conduct PCR for *mecC*, *erm* variants, and other key resistance genes linked to trimethoprim/sulfamethoxazole, penicillin, gentamicin, and ciprofloxacin. This limitation may have led to incomplete or inaccurate resistance profiling, as methods like MIC determination and multiplex PCR are essential for identifying resistance mechanisms and guiding effective therapy [63, 64]. Furthermore, the boiling method applied for DNA extraction may not fully eliminate contaminants such as proteins and lipids, which can affect accuracy [61].

Extended heating can also degrade DNA, reducing sequencing quality [62]. Although practical in our resource‐limited settings, the method requires careful optimization, and commercial extraction kits generally provide a more reliable DNA yield and purity [79, 80]. Most of the AMR data in Malawi comes from urban centers [11, 13, 14, 21–23, 50]. Studies within Africa demonstrate that rural and urban regions can differ significantly in both AMR and *S. aureus spa* typing [66,81,82]. Characterizing regional strains in low‐resource settings is essential for informed public health policy [65]. Future studies are needed to broaden our understanding of AMR in Malawi and to support evidence‐based updates to national treatment guidelines on staphylococcal infections.

## 5. Conclusions

This study emphasizes the value of molecular surveillance programs in rural, resource‐limited regions such as Southern Malawi. Through molecular techniques, we identified multidrug‐resistant staphylococci with diverse *spa* types to improve our regional understanding of staphylococcal epidemiology and support clinical management.

## Author Contributions

W.T., M.T.S., and B.M.H. developed the study concept and design. B.M.H. secured funding for the study. W.T. performed preliminary biochemical tests. W.T. carried out conventional PCR. W.T. sequenced the *spa* gene. W.T. and M.T.S. performed bioinformatics analysis. W.T. conducted data analysis. W.T. prepared the initial manuscript, which was completed and edited by M.P. All authors participated in manuscript revision, reviewed the results.

## Funding

This study was supported by the African Centre of Excellence for Diseases of Huma (P151847).

## Disclosure

All authors approved the final version of the manuscript.

## Ethics Statement

This study was reviewed and approved by the National Health Sciences Research Committee, The Ministry of Health, Malawi, P.O. Box 30277, Lilongwe 3, Malawi. Protocol #22/08/2966. Study samples from Malamulo Hospital were collected from patients who were treated following standard policies and guidelines. Malamulo Hospital IRB approved the use of the isolates and laboratory information system.

## Conflicts of Interest

The authors declare no conflicts of interest.

## Data Availability

All relevant *spa* repeat region sequences were submitted to GenBank with Accession Number BankIt2943795, Sequences PV440593 to PV440600.
